# Fenofibrate Nano-Eyedrops Ameliorate Retinal Blood Flow Dysregulation and Neurovascular Coupling in Type 2 Diabetic Mice

**DOI:** 10.3390/pharmaceutics14020384

**Published:** 2022-02-09

**Authors:** Junya Hanaguri, Noriaki Nagai, Harumasa Yokota, Akifumi Kushiyama, Masahisa Watanabe, Satoru Yamagami, Taiji Nagaoka

**Affiliations:** 1 Department of Visual Science, Division of Ophthalmology, Nihon University School of Medicine, Itabashi-ku, Tokyo 173-8610, Japan; locksteady@icloud.com (J.H.); atokoy18@gmail.com (H.Y.); masahisa_watanabe12-1@yahoo.co.jp (M.W.); yamagami.satoru@nihon-u.ac.jp (S.Y.); 2 Department of Pharmaceutical Sciences, Faculty of Pharmacy, Kindai University, Higashi-Osaka 577-8502, Osaka, Japan; nagai_n@phar.kindai.ac.jp; 3 Department of Pharmacotherapy, Meiji Pharmaceutical University, Kiyose, Tokyo 204-8588, Japan; kushiyama@my-pharm.ac.jp

**Keywords:** neurovascular coupling, diabetic retinopathy, nano-eyedrop, drug delivery, glial function, fenofibrate

## Abstract

We investigated the effect of fenofibrate nano-eyedrops (FenoNano) on impaired retinal blood flow regulation in type 2 diabetic mice. Six-week-old db/db mice were randomly divided into an untreated group (*n* = 6) and treated group, which received FenoNano (*n* = 6). The longitudinal changes in retinal neuronal function and blood flow responses to systemic hyperoxia and flicker stimulation were evaluated every 2 weeks in diabetic db/db mice treated with FenoNano (*n* = 6) or the vehicle (*n* = 6) from ages 8–14 weeks. The retinal blood flow was assessed using laser speckle flowgraphy. We also evaluated the expressions of vascular endothelial growth factor (VEGF), glial fibrillary acidic protein (GFAP), and aquaporin 4 (AQP4) and the phosphorylation of peroxisome proliferator-activated receptor alpha (PPAR-α) by immunofluorescence. In db/db mice treated with FenoNano, both responses were restored from 8 to 14 weeks of age compared with the diabetic mice treated with the vehicle. At 14 weeks of age, the impaired regulation of retinal blood flow during systemic hyperoxia and flicker stimulation improved to about half of that in the db/db mice treated with FenoNano compared with the db/m control group (*n* = 5). FenoNano prevented the activation of VEGF and GFAP expression and increased the AQP4 expression and the phosphorylation of PPAR-α detected by immunofluorescence compared with the diabetic mice treated with the vehicle eyedrop. Our results suggested that the fenofibrate nano-eyedrops prevent retinal glial dysfunction via the phosphorylation of PPAR-α and improves the retinal blood flow dysregulation in type 2 diabetic mice.

## 1. Introduction

Diabetic retinopathy (DR) is a leading cause of preventable blindness and a frequent complication of diabetes. However, treatments are currently only available for the advanced stages of DR and can cause significant adverse effects. Therefore, there is a need for new pharmacological treatments that are effective in the early stages of DR. Fenofibrate is a peroxisome proliferator-activated receptor alpha (PPAR-α) agonist and a widely used triglyceride-lowering medicine. In addition, emerging evidence suggests that fenofibrate provides a broad range of beneficial effects on diabetic complications. Two landmark clinical trials have brought fenofibrate back to the forefront of attention: The Fenofibrate Intervention and Event Lowing in Diabetes (FIELD) [1] and the Action to Control Cardiovascular Risk in Diabetes (ACCORD) [2] previously reported that oral administration of fenofibrate could delay the progression of DR and reduce retinal laser intervention in patients with DR. In addition, many animal model studies have also reported the beneficial effects of the oral administration of fenofibrate on DR [3,4]. Despite these benefits shown, careful attention should be paid to skeletal muscle injury if hypolipidemic drugs, including fenofibrate, are administered systemically. One rare, serious, and potentially fatal side effect to be considered is fenofibrate monotherapy-induced rhabdomyolysis, which is often associated with acute renal failure and myoglobinuria [5]. Therefore, when treating DR, delivery of fenofibrate to the retina using another technique is expected to prevent the myotoxicity of fenofibrate.

Topical administration of an eyedrop is an alternative tool to deliver the drug to the eye, but it is challenging to deliver the drug to the retina. Recent reports have demonstrated significantly improved delivery regarding corneal penetration when using a formulation containing nanoparticles [6,7]. We have also prepared dispersions containing solid nanoparticles that provide high-quantity dispersions containing drug nanoparticles and can be administered easily [8,9]. Moreover, in a previous study using db/db mice, we demonstrated that the impairment of the retinal blood flow regulation in response to systemic hyperoxia and flicker stimulation precedes retinal neuronal dysfunction [10]. Therefore, early detection of retinal flow dysregulation and prompt treatment may protect retinal tissue and prevent or slow the development of irreversible retinopathy. In the present study, we investigated the effect of fenofibrate nano-eyedrops (FenoNano) on impaired retinal blood flow regulation in type 2 diabetic mice.

## 2. Materials and Methods

### 2.1. Animal Preparation

The Ethical Committees of Nihon University and Kindai University Faculty of Pharmacy Committee Guidelines for the Care of Laboratory Animals in accordance with the principles of the Association of Research in Vision and Ophthalmology approved the animal experiments in the current study.

One week before the experiment, 5-week-old male C57BL/KsJ-db/db mice (BKS.Cg-Dock7^m^ +/+ Lepr^db^/J; *n* = 12) and 13-week-old male db/m (congenic nondiabetic littermates, *n* = 6) control mice were purchased from Charles River Laboratories Japan, Inc. (Yokohama, Japan). Japanese albino rabbits (*n* = 10) weighing approximately 2.7 kg were provided by Shimizu Laboratory Supplies Co., Ltd. (Kyoto, Japan). The mice were housed in a temperature-controlled room with a 12-h dark and light cycle and free access to food and water.

The animals were anesthetized with inhaled 2% isoflurane (Pfizer, Tokyo, Japan) using a constant flow rate of 1.5 L/min during the experiment. A heated blanket maintained the rectal temperature from 37 °C to 38 °C. Pupils were dilated with 0.5% tropicamide (Santen Pharmaceutical Co., Osaka, Japan). Blood glucose concentrations were measured from the tail vein (glucose assay kit; Abbott Laboratories, Abbott Park, IL, USA).

### 2.2. Preparation of the Ophthalmic Formulation Containing Fenofibrate Nanoparticles

The ophthalmic formulation was prepared as reported previously [11]. Fenofibrate was obtained from Sigma-Aldrich (St. Louis, MO, USA). Mixtures containing commercially available fenofibrate powder (microparticles), methylcellulose (MC), benzalkonium chloride (BAC), and mannitol were treated with a bead mill (3000 rpm, 30 s, 4 °C) in a tube with 2-mm zirconia beads; the mixtures were added to saline containing 2-hydroxypropyl-β-cyclodextrin (HPβCD). The dispersions with 0.1-mm zirconia beads then were crushed with the bead mill (5500 rpm, 30 s × 15 times, 4 °C) to produce the fenofibrate nanoparticles (FenoNano) (Supplemental Figure S1). The ophthalmic formulation containing fenofibrate microparticles (Feno-Micro) was prepared by dispersing fenofibrate powder into saline containing MC, BAC, mannitol, and HPβCD. The ophthalmic formulations were comprised of 2% fenofibrate and the vehicle (0.5% MC, 5% HPβCD, 0.005% BAC, and 0.5% mannitol).

### 2.3. High-Performance Liquid Chromatography (HPLC)

The fenofibrate concentration was determined by HPLC using a Shimadzu LC-20AT system (Kyoto, Japan) with an Inertsil ODS-3 column (GL Science, Tokyo, Japan) (Supplemental Figure S2). The mobile phase was 20-mM potassium phosphate solution/acetonitrile (3:7, *v*:*v*), with a flow rate of 0.25 mL/min. The fenofibrate was detected at 254 nm at 35 °C.

### 2.4. Measurement of Fenofibrate Ophthalmic Formulation Characteristics

The particle size was measured using the SLDA-7100 (Shimadzu Corp., Kyoto, Japan) and a NANOSIGHT LM10 (Quantum Design Japan, Tokyo, Japan), and atomic force microscopy images were obtained using the SPM-9700 (Shimadzu Corp., Kyoto, Japan) [12].

### 2.5. Measurement of Fenofibrate Concentration in the Retina of Mice

The ophthalmic formulation (30 µL) was administered to the right eye (instilled eye). The mice were killed by a lethal injection of pentobarbital, and the retinas of the instilled eye and the left untreated eye were collected, homogenized in methanol on ice, and centrifuged at 9100× *g* for 15 min at 4 °C. The fenofibrate levels in the supernatants were determined by HPLC.

### 2.6. In Vitro Transcorneal Penetration of Fenofibrate Ophthalmic Formulations

The transcorneal penetration study was performed as we reported previously [12]. Rabbits were killed by pentobarbital injected into the marginal ear vein. The corneas were carefully removed and placed on a methacrylate cell at 35 °C. We filled the donor chamber exposed to the exterior corneal surface with the fenofibrate ophthalmic formulation, and the reservoir chamber was filled with 10-mM HEPES buffer (pH 7.4). Fifty microliters of the samples were withdrawn from the reservoir chamber at the indicated times and replaced with the same volume of buffer, and the fenofibrate concentration in the samples was measured by HPLC.

### 2.7. Measurement of Fenofibrate Concentration in the Blood and Ophthalmic Tissue of Rabbits

FenoNano (30 µL) was instilled into the right eyes of the rabbits twice daily for seven days. One hour after the last administration, the rabbits were killed by pentobarbital, and the blood, cornea, lens, vitreous body, sclera, choroid, and retina were collected. These samples were homogenized in methanol on ice and centrifuged at 9100× *g* for 15 min at 4 °C. The fenofibrate levels in the supernatants were determined by the HPLC.

### 2.8. Longitudinal Topical Administration Study Protocol

#### 2.8.1. Intraocular Pressure (IOP) and Systemic Blood Pressure (BP) Measurements

Systemic BP and IOP were measured 30 min after induction of anesthesia. The BP was measured at the tail with an automatic sphygmomanometer (THC-31, Softron, Tokyo, Japan). the IOP was measured using a handheld tonometer (TonolabTV02, ME Technical, Tokyo, Japan). The mean arterial BP (MABP) was derived from systolic (SBP) and diastolic (DBP) BPs using the standard formula: MABP = DBP + (SBP − DBP)/3. During the experiments, mice were kept in a prone position, and therefore, the ocular perfusion pressure (OPP) was calculated using the formula: OPP = MABP − IOP [13].

#### 2.8.2. Measurement of Retinal RBF

RBF was measured with the LSFG-micro system (Softcare Co., Ltd., Fukutsu, Japan) designed for small animals [13]. The LSFG, which uses the same principle as the LSFG-micro, has been used to quantitatively estimate the ocular (optic nerve head [ONH], choroid, and retina) blood flow in humans [14] and animals [13]. The principle of the LSFG system is explained elsewhere [13]. Briefly, a mean blur rate (MBR) is generated from blurring the speckle pattern produced by the backscattered light of the coherent laser caused by the flow of blood cells. The MBR obtained from the vascular area at the ONH reflects the entire retinal circulation and can be used as an index of the RBF [13]. LSFG analyzer software (version 3.2.19.0, Softcare Co., Ltd., Fukutsu, Japan) analyzed the average vessel MBR. The MBR outcomes in response to hyperoxia and flicker light stimulations were expressed as the percentage of change from baseline.

#### 2.8.3. Induction of Hyperoxia

A baseline value was obtained before hyperoxia began using the mean of three consecutive flow measurements at 1-min intervals for 3 min. Systemic hyperoxia was induced in the mice by providing 10-min inhalation of 100% oxygen, as described previously [10,13]. The RBF was measured every minute for 20 min (10-min each of stimulation during hyperoxia and recovery after systemic hyperoxia termination) [13].

#### 2.8.4. Induction of Flicker Stimulation

The mice were dark-adapted for 2 h in ambient light reduced to 1 lux or less. flicker light stimulation was provided for 3 min with a light intensity of 30 lux for the rod-dominant mouse retina [13]; 12-Hz flicker stimulation triggered a maximal RBF response [13]. The RBF was measured at 20-s intervals during and after flicker light stimulation for 6 min (3-min each of stimulation and recovery). The baseline RBF was obtained using the mean of three consecutive flow measurements obtained over 1 min (20-s intervals) before flicker light stimulation started.

### 2.9. Measurement of RBF in Response to Systemic Hyperoxia and Flicker Stimulation

Thirty microliters of 2% FenoNano (*n* = 6) or the vehicle (*n* = 6) were instilled twice daily (7:00 a.m., 7:00 p.m.) into the right eyes of 6-week-old db/db mice for 8 weeks. Longitudinal assessments of the RBF in each animal were done on two consecutive days every 2 weeks from 8 to 14 weeks of age (day 1, response of the RBF to systemic hyperoxia was measured; day 2, the response to flicker light stimulation was performed). An independent masked observer (AK) performed all data calculations and analyses.

We confirmed previously that the systemic BP, IOP, and OPP are unaffected by hyperoxia or flicker light stimulation in mice [13].

### 2.10. Immunohistochemistry

After the measurements, all animals were euthanized, and the eyeballs were extracted. Sternotomy was performed under systemic anesthesia with 3% isoflurane; normal saline was perfused into the left ventricle to wash out the circulating blood, perfusion of 4% paraformaldehyde (PFA) followed immediately, and the eyeballs were enucleated, stored overnightin 4% PFA at 4 °C, andafter two washings in phosphate-buffered saline embedded in a Tissue-Tek OCT Compound (Sakura Finetek Japan, Tokyo, Japan) and stored at −80 °C until analysis.

Ten-micrometer-thick sections were cut with a cryostat (HM505, Microm, Walldorf, Germany), stained with isolectin B4 (dilution, 1:400; #I21413 Thermo Fisher Scientific, Waltham, MA, USA) and the primary antibodies against phospho-PPAR-α (dilution, 1:200; #ab3484 Abcam, Cambridge, MA, USA), AQP4 (dilution, 1:200; #A5971 Sigma-Aldrich, St. Louis, MO, USA), glial fibrillary acidic protein (GFAP; Ready to use, Dako, Glostrup, Denmark), and VEGF (1:100, #07-1420 Sigma-Aldrich) overnight at 4 °C, and incubated with the secondary donkey anti-rabbit IgG (H+L) Alexa Fluor 488 (dilution, 1:400; Thermo Fisher Scientific) for 2 h at room temperature. Albumin was immunohistochemically stained with the primary antibody (dilution, 1:200; #ab8940 Abcam) and incubated with an Alexa Fluor 594-conjugated anti-sheep IgG antibody (dilution, 1:400; Thermo Fisher Scientific). Immunofluorescent images were obtained using the FluoView 1000 confocal microscope (Olympus, Tokyo, Japan) and BZ-9000 microscope (Keyence, Osaka, Japan). The fluorescein intensities were calculated using ImageJ software. Briefly, the region of interest, defined as the layers between the inner plexiform layer (IPL) and outer nuclear layer (ONL), was determined using a freehand selection tool. The mean gray values were used to compare the average fluorescein intensity among the groups.

### 2.11. Statistical Analysis

Data were expressed as the mean ± standard error of the mean; the number of animals studied was represented by *n*. Changes in the RBF were calculated as the percentage change from the baseline. The Kolmogorov-Smirnov test was used to assess the normality of the data distribution. For statistical analysis, one-way or two-way repeated measures analysis of variance (ANOVA) followed by Dunnett’s test or Holm-Sidak test, respectively, determined the significance of the experimental intervention across different time points within and between groups, as appropriate (Prism 9; GraphPad Software, San Diego, CA, USA). A *p*-value < 0.05 was considered statistically significant.

## 3. Results

### 3.1. Changes in Fenofibrate Concentrations in the Rabbits Instilled with Fenofibrate Ophthalmic Formulations

**Figure 1:**
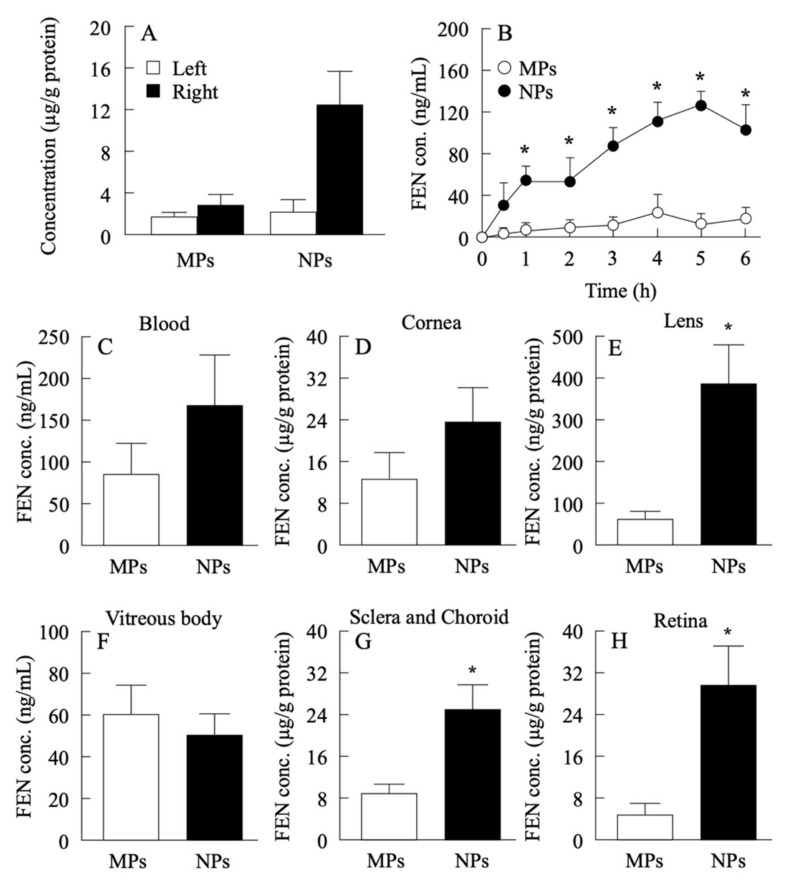
Changes in fenofibrate concentrations in mice and rabbits instilled with fenofibrate ophthalmic formulations. (**A**) Fenofibrate concentrations in the retina of the right eye (instilled eye) and left eye (untreated eye) of mice 60 min after 1 instillation of 5 µL of 2% FenoNano (NPs) or 2% Feno-Micro (MPs). (**B**) In vivo transcorneal penetration of FenoNano and Feno-Micro in rabbit eyes. (**C**–**H**) Changes in the concentration of fenofibrate in the blood (**C**), cornea (**D**), lens (**E**), vitreous body (**F**), sclera and choroid (**G**), and retina (**H**) of rabbit eyes instilled with FenoNano (NPs) or Feno-Micro (MPs). *n* = 4 to 5. * *p* < 0.05 vs. Feno-Micro (MPs) for each category. FEN conc: concentration of fenofibrate.

First, we confirmed that novel ophthalmic formulations containing fenofibrate nanoparticles (Supplemental Figure S1) can deliver fenofibrate into the retina in normal mice and rabbits. After one eyedrop of 2% FenoNano, the fenofibrate concentrations in the right eye (instilled eye) increased significantly compared with those in the left eye (untreated eye) of the same mouse instilled once with FenoNano and both eyes of other mice instilled with Feno-Micro (Figure 1A). In vivo transcorneal penetration of FenoNano and Feno-Micro in rabbit corneas showed that the concentration of fenofibrate increased in eyes treated with FenoNano compared with those treated with Feno-Micro from 1 to 6 h after one eyedrop of FenoNano. (Figure 1B). We also observed an increased concentration of fenofibrate in the lens, sclera, choroid, and retina in eyes treated with FenoNano compared with those treated with Feno-Micro. In contrast, the concentrations of fenofibrate did not differ significantly between FenoNano and Feno-Micro in the blood, cornea, and vitreous body (Figure 1C–H).

### 3.2. Longitudinal Assessment of Systemic and Ocular Parameters

**Figure 2:**
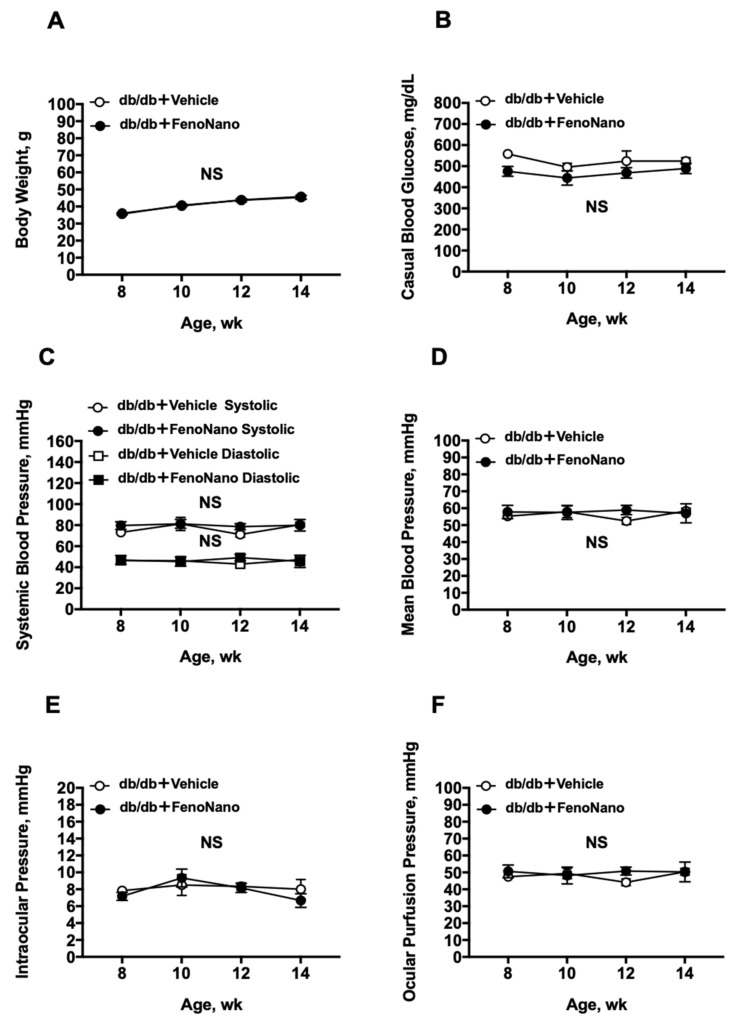
Average systemic and ocular parameters in db/db mice treated with 2% FenoNano eyedrops or placebo from 8 to 14 weeks of age. There were no significant differences in Body weight (**A**), Casual blood glucose (**B**), Systemic blood pressure (**C**), Mean blood pressure (**D**), Intraocular pressure (**E**), and Ocular perfusion pressure (**F**) between the two groups from 8 to 14 weeks of age. NS = not significant between groups and within groups.

During the experiment, no significant differences were found in body weight, blood glucose level, systemic blood pressure (BP), intraocular pressure (IOP), and ocular perfusion pressure (OPP) between the untreated db/db mice and db/db mice treated with FenoNano (two-way repeated measures ANOVA; Figure 2).

### 3.3. Longitudinal Assessment of Resting Retinal Blood Flow in Diabetic Mice

**Figure 3:**
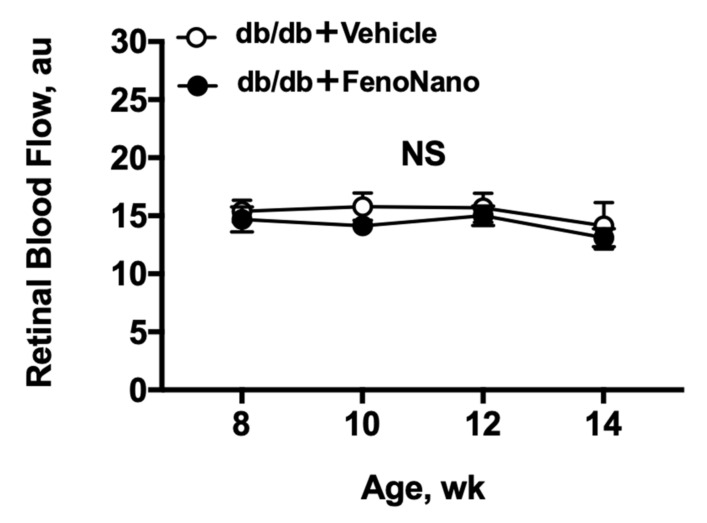
Time course of the changes in the retinal blood flow in db/db mice treated with 2% FenoNano eyedrops (*n* = 6) or placebo (*n* = 6) from 8 to 14 weeks of age. No significant changes in retinal blood flow (au) were observed in both groups throughout by one-way repeated measures ANOVA (*p* = 0.46 for db/db with FenoNano and *p* = 0.83 for db/db with placebo). There was also no difference in the changes in the resting retinal blood flow from 8 to 14 weeks of age between two groups (two-way repeated measures ANOVA). NS = not significant between groups and within groups.

There were no significant changes in the resting retinal blood flow in both untreated db/db mice and db/db mice treated with FenoNano exhibited from 8 weeks to 14 weeks of age, with no differences between the groups (two-way repeated measures ANOVA; Figure 3).

### 3.4. Longitudinal Assessment of Retinal Blood Flow in Response to Systemic Hyperoxia in Diabetic Mice

**Figure 4:**
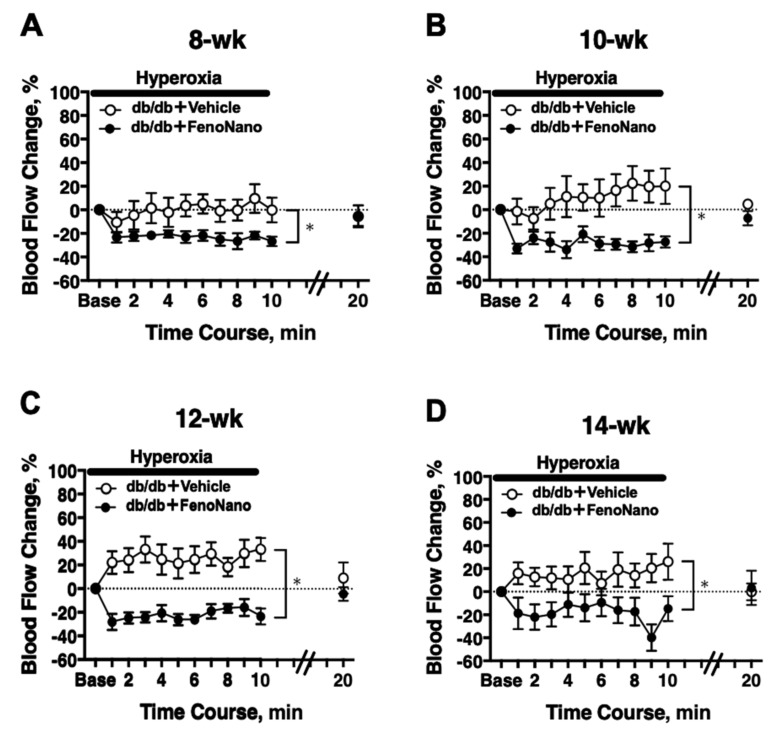
The time course of the changes in retinal blood flow in response to systemic hyperoxia in db/db mice with placebo (*n* = 6) or 2% FenoNano (*n* = 6) at 8 (**A**), 10 (**B**), 12 (**C**) and 14 (**D**) weeks old. There was a significant difference in the changes in blood flow during hyperoxia between the db/db mice treated with a placebo and those treated with the FenoNano eyedrops for each age studied (two-way repeated measures ANOVA). * *p* < 0.05 between groups. Solid bar = period of hyperoxia.

In 8-week-old db/db mice, there was a significant difference in the hyperoxia-induced changes in the retinal blood flow between the untreated db/db mice and the db/db mice treated with FenoNano (two-way repeated measures ANOVA; Figure 4A). Significant differences in the hyperoxia-induced flow changes were also observed at 10, 12, and 14 weeks of age (Figure 4B–D).

### 3.5. Longitudinal Assessment of Retinal Blood Flow in Response to Flicker Stimulation in Diabetic Mice

**Figure 5:**
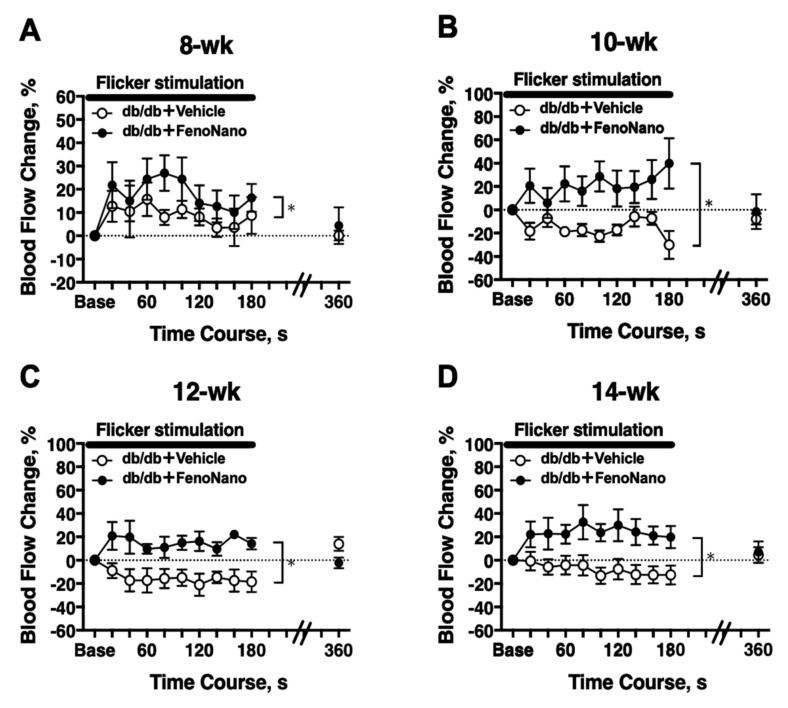
The time course of the changes in retinal blood flow in response to flicker stimulation in db/db mice treated with the 2% FenoNano eyedrops (*n* = 6) or placebo (*n* = 6) at 8 (**A**), 10 (**B**), 12 (**C**) and 14 (**D**) weeks old. There were significant differences in the changes in blood flow during flicker stimulatio between db/db mice treated with the placebo and 2% FenoNano for each age studied (two-way repeated-measures ANOVA). * *p* < 0.05 between groups. Solid bar = period of flicker stimulation.

In 8-week-old db/db mice, there was a significant difference in the flicker-induced change in the retinal blood flow between untreated db/db mice and db/db mice treated with FenoNano (two-way repeated measures ANOVA; Figure 5A). Significant differences in the flicker-induced flow changes were also observed at 10, 12, and 14 weeks of age (Figure 5B–D).

### 3.6. Maximum Retinal Blood Flow Change in Response to Hyperoxia and Flicker Stimulation at 14 Weeks in Diabetic Mice and db/m Nondiabetic Control Mice

**Figure 6:**
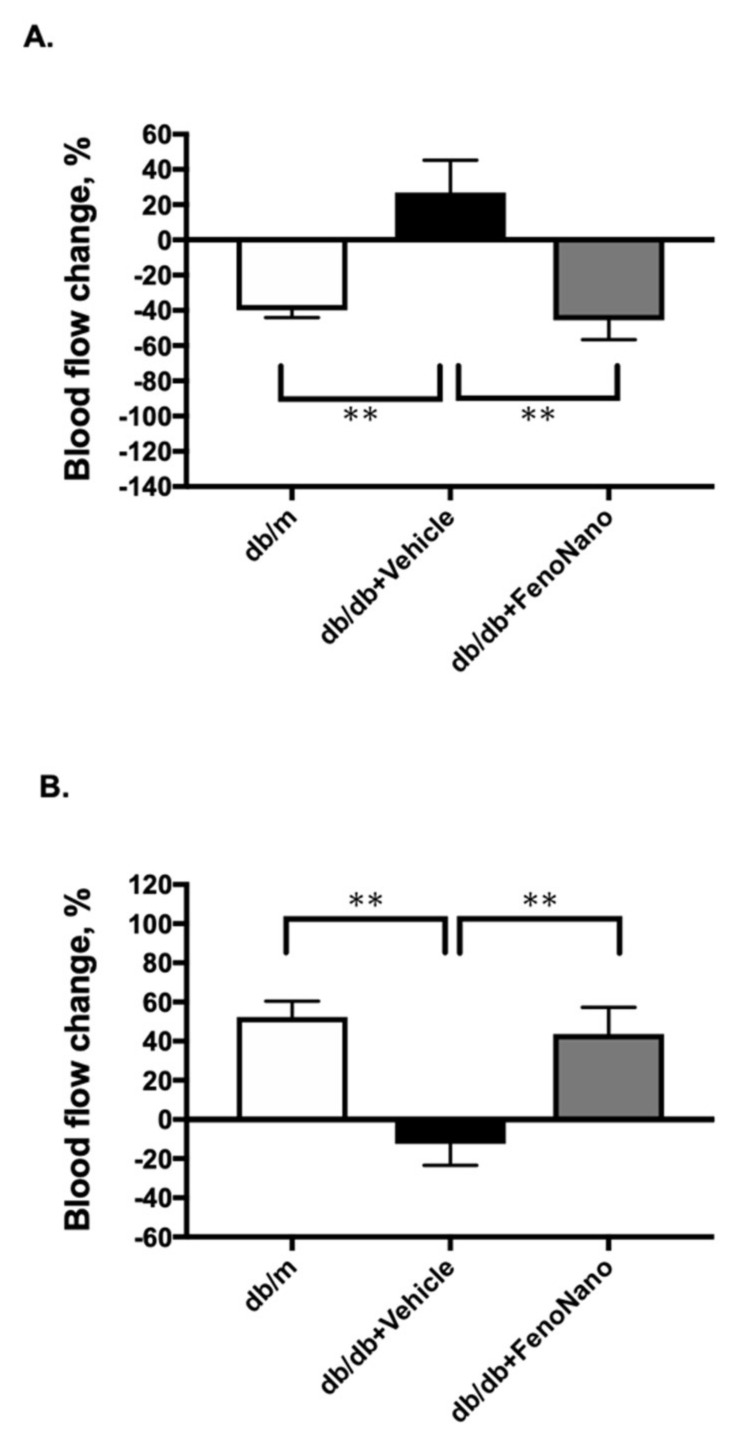
The maximal changes in retinal blood flow from the baseline in response to hyperoxia (**A**) and flicker stimulation (**B**) in 14week-old db/m mice (*n* = 5) as the nondiabetic control and db/db mice treated with the placebo (*n* = 6) and the 2% FenoNano eyedrops (*n* = 6). ** *p* < 0.01 compared with db/m by one-way ANOVA, followed by Dunnett’s test.

At 14 weeks of age, the same protocol for systemic hyperoxia and flicker stimulation was performed in the two diabetic mice and db/m control mice groups (Figure 6). We previously confirmed that the retinal blood flow response to both stimulations remained stable in db/m nondiabetic control mice from 8 to 20 weeks of age [10]. Retinal blood flow measurements were performed only once at 14 weeks of age, in line with the animal welfare criteria for our institution. Figure 5 shows that the changes in retinal blood flow observed in the control db/m mice in response to both systemic hyperoxia (A) and flicker stimulation (B) were abolished in db/db treated with placebo mice but were restored to about half of the decrease in the retinal blood flow in db/db mice treated with FenoNano compared to the db/m control group.

### 3.7. Beneficial Effect of Topical Administration of the Fenonano Eyedrop on GFAP and VEGF Expression

**Figure 7:**
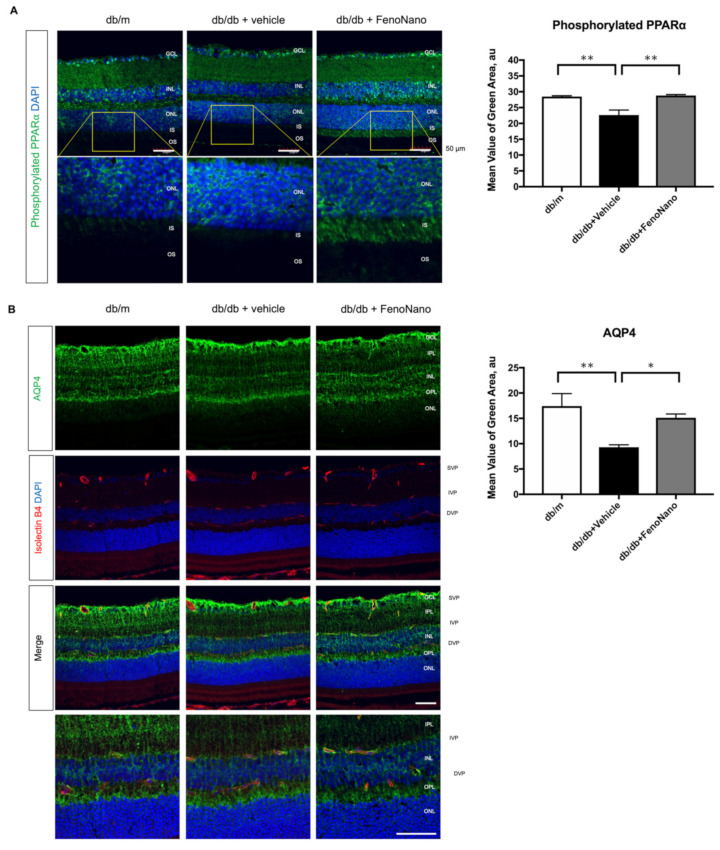

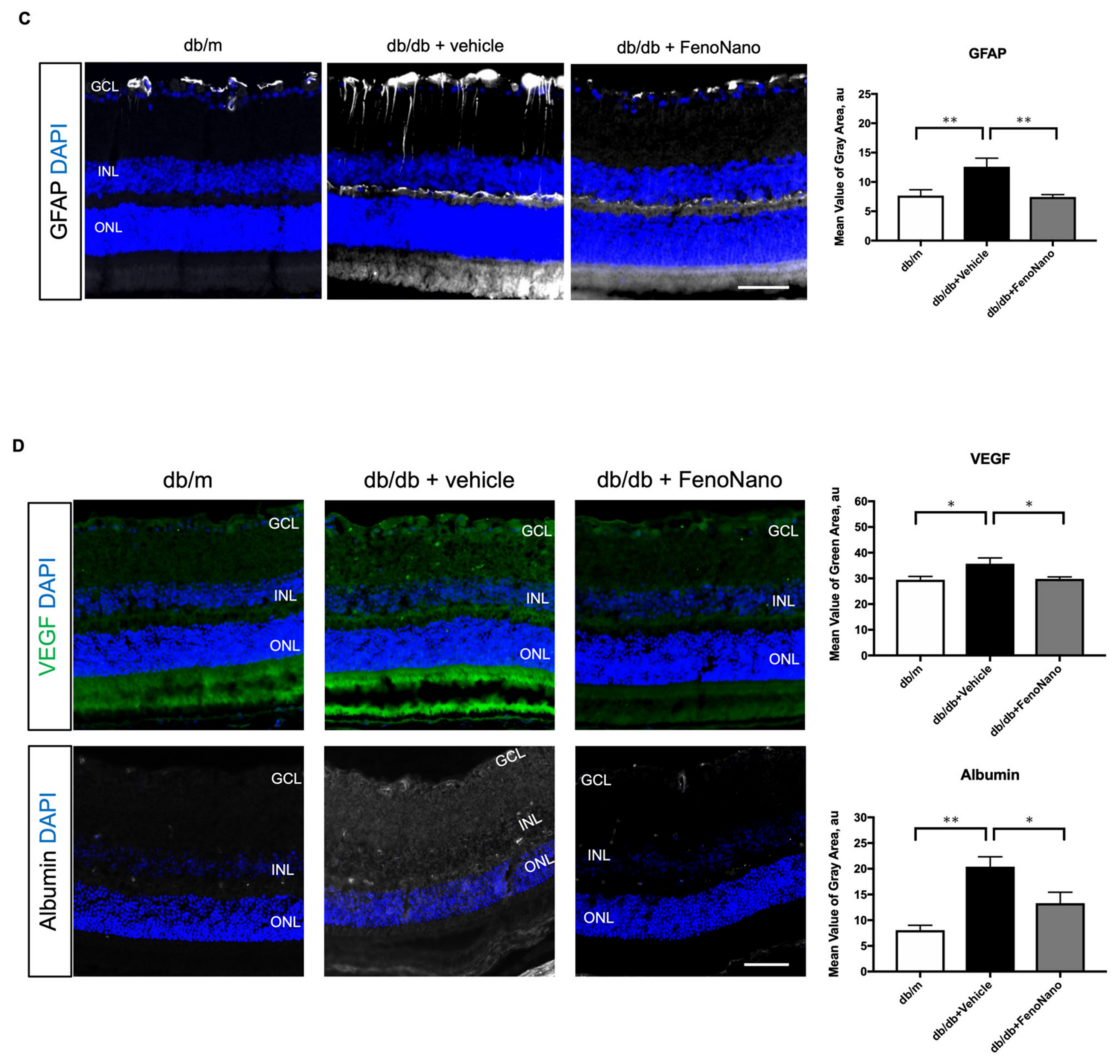
The 2% Fenofibrate eyedrops (FenoNano) modulate diabetes-induced alterations of molecules in the retina of db/db mice. (**A**) Immunohistochemical analysis of the effect of FenoNano on the expression of phosphorylated PPAR-α (green) in the retina of db/db mice (Scale bar: 50 µm). (**B**) Immunohistochemical analysis of the effect of FenoNano on the expression of aquaporin 4 (AQP4) (green) in the retina of db/db mice. The blood vessels were stained with isolectin B4 (red). (**C**) Immunohistochemical analysis of the effect of FenoNano on gliosis in db/db mice. Glial fibrillary acidic protein (GFAP) (grey) is a marker of gliosis. (**D**) Immunohistochemical analysis of the effect of FenoNano on vascular leakage in the retinas of db/db mice. The expression of VEGF (green) and albumin (grey) leakage were visualized. The nuclei were counterstained with DAPI (blue). There were significant differences in the fluorescein intensities of phosphorylated PPAR-α (*p* = 0.0004), AQP4 (*p* = 0.0052), GFAP (*p* = 0.0048), VEGF (*p* = 0.018), and albumin (*p* = 0.006) among the three groups by one-way ANOVA. The region of interest for fluorescent quantification was defined as the layers between the inner plexiform layer (IPL) and outer nuclear layer (ONL). There were significant decreases in the fluorescein intensities of phosphorylated PPAR-α and AQP4 and increases in the intensities of GFAP, VEGF, and albumin in the untreated db/db mice compared with the nondiabetic db/m mice, but the increased intensities of phosphorylated PPAR-α and AQP4 decreased significantly, reducing the decreased intensities of GFAP, VEGF, and albumin, which were significantly ameliorated in the FenoNano-treated murine retinas. * *p* < 0.05, ** *p* < 0.01 by one-way ANOVA, followed by Dunnett’s test. GCL, ganglion cell layer; IPL, inner plexiform layer; INL, inner nuclear layer; ONL, outer nuclear layer; OPL, outer plexiform layer; IS, inner segment; OS, outer segment; SVP, superficial vascular plexus; IVP, intermediate vascular plexus; DVP, deep vascular plexus (Scale bar: 50 µm).

To clarify the beneficial effect of FenoNano on the pathogenesis of DR, we performed an immunohistochemical analysis to investigate whether the long-term systemic administration of FenoNano ameliorated glial activation and hyperpermeability in the retina of the db/db type 2 diabetic mice model. We stained the murine tissue for phosphorylated PPAR-α (the target of fenifibrate), AQP4, GFAP (Muller cell function), VEGF, and albumin (vascular leakage). As shown in Figure 7, there were significant differences in the fluorescence intensities of phosphorylated PPAR-α (*p* = 0.0004), AQP4 (*p* = 0.0052), GFAP (*p* = 0.0048), VEGF (*p* = 0.018), and albumin (*p* = 0.006) among the three groups by one-way ANOVA. FenoNano significantly increased the expression of phosphorylated PPAR-α (Figure 7A) and AQP4 (Figure 7B) and decreased the expression of GFAP (Figure 7C) and VEGF (Figure 7D) in the retina of db/db mice compared with the untreated db/db mice. Albumin leakage was significantly observed in all the retinal layers of the db/db + vehicle mice compared to the db/m mice. In the db/db + FenoNano mice, extraluminal staining of albumin was highly abolished (Figure 7D). The FenoNano treatment ameliorated these changes, significantly increasing the fluorescence intensity of phosphorylated PPAR-α and AQP4 and decreasing the fluorescence intensity of GFAP, VEGF, and albumin staining.

## 4. Discussion

The current longitudinal interventional study revealed that the fenofibrate nano-eyedrops (FenoNano) prevent glial dysfunction via the phosphorylation of PPAR-α and improve the retinal blood flow dysregulation and impaired neurovascular coupling in the retina in type 2 diabetic mice.

We found that one drop of FenoNano immediately increased the fenofibrate concentration in the instilled eye and kept increasing it until 6 h compared with the control eyedrop with commercially available fenofibrate powder (Feno-Micro) (Figure 1A,B). The concentration of fenofibrate also increased in the lens, sclera, and choroid but did not significantly change in the blood, cornea, and vitreous body (Figure 1C–H). These results suggest that the fenofibrate nanoparticles reached the retina, probably via the periocular and/or uveoscleral route [15]. In comparison, Chen et al. reported that a fenofibrate intravitreal injection maintained the active drug levels less than 1 week in diabetic mice [16]. Although the intravitreal injection technique is used widely to deliver the drug to the retina, it is also associated with complications, including iatrogenic cataract, retinal detachment, vitreous hemorrhage, and endophthalmitis. Therefore, the current results indicate that FenoNano eyedrops are desirable to noninvasively deliver fenofibrate to the retina.

It is important to consider the possible effects of fenofibrate in the blood after eyedrop instillation to prevent severe systemic side effects. As shown in Figure 1C, the concentration of fenofibrate increased in the blood. However, the concentrations of fenofibrate in the blood (100 ng/mL) after one drop of both FenoNano and Feno-Micro were much lower than that in the blood after oral administration of fenofibrate (10 µg/mL) in healthy volunteers [17]. Moreover, because humans have a larger blood volume compared to rabbits and mice, the systemic exposure may be even lower in humans. Therefore, our data support our hypothesis that we can minimize the systemic side effect of fenofibrate by using FenoNano eyedrops.

No in vivo study has examined the effect of fenofibrate on the regulation of retinal blood flow. Previously, we found that fenofibrate dilated the isolated porcine retina arterioles via the production of nitric oxide from the vascular endothelium using an in vitro isolated vessel technique [18]. However, only the acute effect of fenofibrate was evaluated in the previous study, so the long-term effect of fenofibrate on retinal blood flow remains unclear. We found that the long-term topical administration of FenoNano eyedrops can restore the dysfunction of the retinal blood flow in response to flicker stimulation, which is an index of retinal neurovascular function [19]. The small but significant difference in retinal blood flow during the flicker stimulation between the placebo and treated groups in 8-week-old animals (*p* = 0.002, Figure 5A) suggests that even a short two-week treatment of FenoNano eyedrops may be enough to restore the impaired retinal neurovascular coupling in diabetic mice. In addition, the beneficial effects of long-term topical administration of FenoNano on retinal neurovascular coupling were apparently confirmed in animals 10–14 weeks of age (Figure 5B–D).

We previously found that the retinal circulation became unresponsive to both the stimulation of systemic hyperoxia and flicker stimulation (10–12 weeks), and the flow responses tended to reverse in the later stages of diabetes (14–20 weeks) [10], which we observed in untreated db/db mice in response to systemic hyperoxia (Figure 4 and Figure 6A) and flicker stimulation (Figure 5 and Figure 6B). Although we could not examine the exact mechanisms underlying the reversed flow responses to both stimulations, the observed impairment of the flow response to systemic hyperoxia might be due to the diminished vascular responsiveness to ET-1 [20], which may be related to ET_A_ receptor desensitization [21].

The involvement of PPAR-α in the beneficial effect of the systemic administration of fenofibrate on diabetic retinas remains controversial [22,23,24]. Since fenofibrate can modulate the activation of PPAR-α [22], a phosphoprotein [25], we also examined the expression of phosphorylated PPAR-α in the retina as a target molecule of FenoNano eyedrops. In the current study, we first confirmed that the long-term administration of FenoNano restored the phosphorylation of PPAR-α in murine retinas in db/db mice (Figure 7A). Our pathological results clearly demonstrated that phosphorylated PPAR-α may be involved with the improvement in retinal blood flow dysregulation in response to hyperoxia and flicker stimulation in diabetic mice. Bogdanov et al. reported that the oral administration of high-dose fenofibrate (100 mg/kg/day) for 1 week resulted in a significant decrease in both glial activation and the rate of apoptosis in GCL compared with diabetic mice treated with the vehicle [26]. Although we did not examine the apoptosis in the GCL in the current study, the phosphorylated PPAR-α-positive cells were found in the GCL, as well as the inner nuclear layer (Figure 7A). Since flicker-induced hyperemia in the retina is thought to compensate for an increase in ganglion cell activity, which is dependent on signals from the photoreceptors, bipolar cells, horizontal cells, and amacrine cells [27,28], the increased phosphorylated PPAR-α-positive cells in the GCL may contribute to the improved retinal blood flow response to flicker stimulation in db/db mice treated with FenoNano eyedrops. In addition, the phosphorylated PPAR-α-positive cells were also found in the inner segments in db/db mice treated with FenoNano eyedrops, although hematoxylin and eosin (HE) staining showed that both the inner and outer segments were intact in all mice (Supplemental Figure S3). Since PPAR-α is involved in the regulation of fatty acid and energy metabolism in the mitochondria [29], the increase in phosphorylated PPAR-alpha in the mitochondria-rich inner segments in db/db mice treated with FenoNano eyedrops may contribute to the improved neurovascular coupling in the retina in diabetic mice.

The factors produced from Müller cells can alter the vascular permeability, blood flow, and cell survival processes that surround all retinal blood vessels [30]. Therefore, it is needed to investigate the possible role of these cells in the pathogenesis of retinal microangiopathy. Previous studies have also reported that diabetes provokes the dysfunction of Müller cells [31,32]. In particular, the gliosis (glial activation) of Müller cell occurs early in DR and may be involved in early microvascular dysfunction [33]. Bogdanov et al. reported [26] that the systemic administration of fenofibrate resulted in a significant decrease in Müller cell gliosis, which can be detected by the expression of GFAP compared with type 2 diabetic db/db mice treated with the vehicle. Since PPAR-α is expressed in Müller cells [3], Müller cell dysfunction will be a novel target to treat DR using FenoNano eyedrops. We found that FenoNano eyedrops decrease GFAP expression (Figure 7C), indicating reduced gliosis, in diabetic retinas. The improvement in Müller glial cell function seems to be responsible for the restoration of retinal blood flow dysregulation in response to systemic hyperoxia and flicker stimulation, because the retina glial cells play important roles in both responses [34,35].

It has been reported that Müller cells is a major source of VEGF in the retina in diabetic mice [36]. Furthermore, retinal VEGF derived from Müller cells may change the protein expression and produced the peroxynitration, which play important roles in the pathogenic process of DR via the inflammation, neovascularization, and vascular leakage in the retina. Therefore, it is believe that these cells may be a potential target for the treatment of DR [37]. In addition, there was a previous animal study to report the inhibitory effects of PPAR-α on VEGF expression throughout the retina in a type 1 diabetes rat model [16]. In the current study, we observed that the increased expression of VEGF in untreated db/db mice was reduced in the db/db mice treated with FenoNano eyedrops in accordance with the increased phosphorylation of PPAR-α (Figure 7D), suggesting that FenoNano eyedrops may have a beneficial potential of reducing the VEGF level in the retina, probably via the phosphorylation of PPAR-α in the retina. However, further clinical study is needed to elucidate this possibility.

Since the water channel AQP4 is located at retinal capillaries and contributes to maintaining the water balance in retinal tissue, AQP4 may be associated with the pathogenesis of DR and macular edema via the increased vascular permeability in the diabetic retina [38]. Cui et al. reported that AQP4 knockdown led to an exacerbation of retinopathy, with increased vascular permeability, retinal thickness, and enhanced retinal expression of proinflammatory factors, VEGF, and GFAP in diabetic rats [39]. In the current study, we confirmed that the decrease in AQP4 immunofluorescence in db/db mice improved as the result of the treatment with FenoNano eyedrops in accordance with the reduction of the overexpression of VEGF and increased albumin leakage (Figure 7) [40]. Although the relations between the retinal AQP4 and the phosphorylation of PPAR-α and retinal blood flow regulation remain unclear, the restoration of decreased AQP4 induced by the long-term administration of FenoNano eyedrops may imply potential for the novel treatment of DR and diabetic macular edema.

There are several limitations of this study to consider. First, we did not quantitatively analyze the mRNA levels of PPAR-α in db/db mice with the long-term treatment of FenoNano. Second, in addtion to the small sample size (*n* = 5 to 6), we did not perform a longitudinal follow-up of the db/m nondiabetic control mice, because we previously confirmed that the retinal blood flow in the db/m nondiabetic control mice in the study age range remained stable in response to systemic hyperoxia and flicker stimulation [10] due to the animal welfare criteria in our institution. Third, we could not quantify the expression levels of phosphorylated PPAR-α, AQP4, GFAP, VEGF, and albumin in the retina in our longitudinal observational study. Further studies are needed to determine the relation between the quantitative changes of these molecules detected by Western blot or ELISA and physiological data, including the retinal blood flow responses to flicker stimulations at time points different from 8 to 14 weeks of age. Finally, further studies are also needed to examine the retinal neuronal function by electroretinography in this study to demonstrate the beneficial effect of FenoNano eyedrops on retinal neurovascular coupling in diabetic mice. Finally, further studies are needed to examine retinal neuronal function by electroretinography to demonstrate the beneficial effect of the FenoNano eyedrop treatment on retinal neurovascular coupling in diabetic mice.

Our results suggest that fenofibrate nano-eyedrops prevent retinal glial dysfunction via the phosphorylation of PPAR-α and improve retinal neurovascular coupling in type 2 diabetic mice.

## Data Availability

The data presented in this study are available on request from the corresponding author.

## Supplementary Materials

The following supporting information can be downloaded at: https://www.mdpi.com/article/10.3390/pharmaceutics14020384/s1: Figure S1: Size frequency distribution and images of fenofibrate ophthalmic formulations. Figure S2: The representative HPLC chromatograms to determine the rat retinal concentration of fenofibrate. Figure S3: The HE stainings of the whole retina in non-diabetic control db/m, db/db with vehicle, and db/db with FenoNano eyedrop at 14 weeks of age.
