# Supplementary material for: Fenofibrate Nano-Eyedrops Ameliorate Retinal Blood Flow Dysregulation and Neurovascular Coupling in Type 2 Diabetic Mice
Source: Pharmaceutics. 2022 Feb 9;14(2):384. doi: 10.3390/pharmaceutics14020384 (PMC8876509; doi:10.3390/pharmaceutics14020384)
Supplement: Supplementary file 1 [file pharmaceutics-14-00384-s001.zip › pharmaceutics-1532497-supplementary.pdf]

pharmaceutics

Supplementary Materials: Fenofibrate Nano-eyedrops Ameliorate Retinal Blood Flow Dysregulation and Neurovascular Coupling in Type 2 Diabetic Mice

Junya Hanaguri, Noriaki Nagai, Harumasa Yokota, Akifumi Kushiyama, Masahisa Watanabe, Satoru Yamagami and Taiji Nagaoka

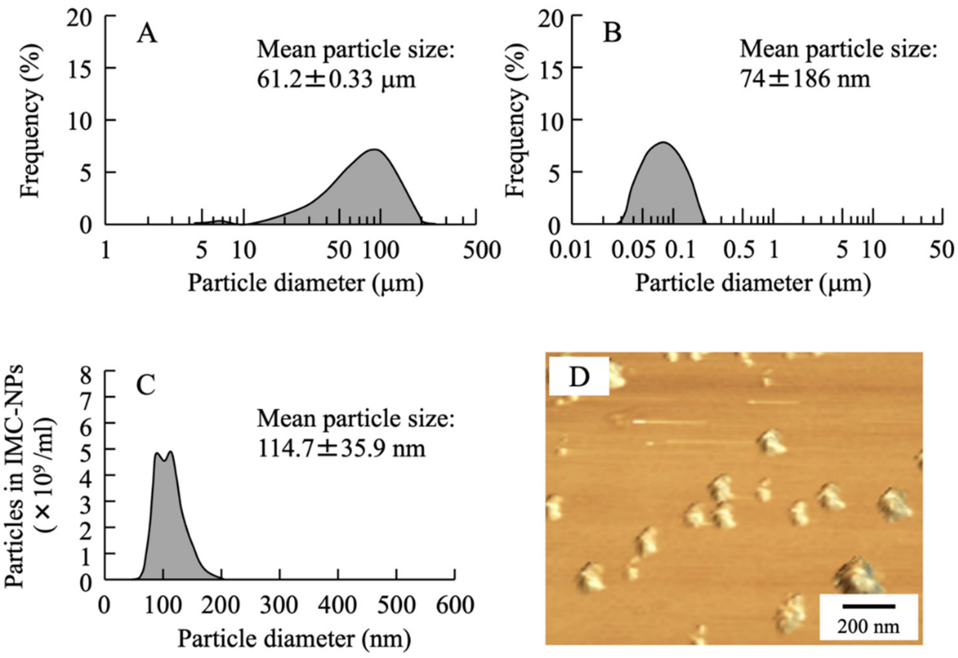

Figure S1. Size frequency distribution and images of fenofibrate ophthalmic formulations. A and B: cumulative size distribution of Feno-Micro (**A**) and FenoNano (**B**) in the SALD-7100. (**C**) cumulative size distribution of FenoNano in the NANOSIGHT LM10. (**D**) AFM image of FenoNano in the SPM-9700.

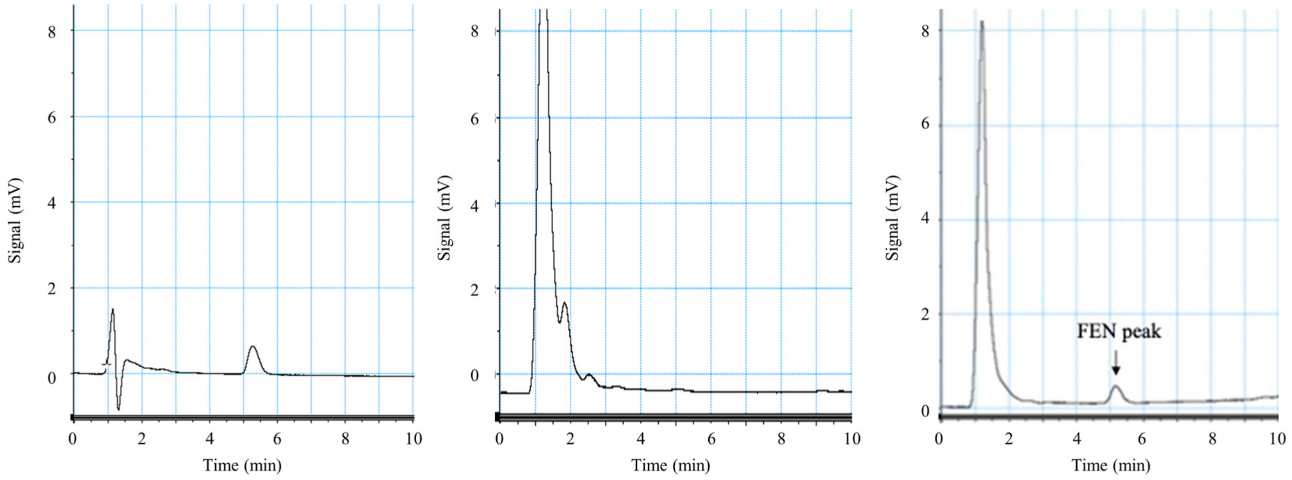

Figure S2. The representative HPLC chromatograms to determine the rat retinal concentration of fenofibrate (FEN). (**A**) HPLC chromatogram of FEN reagent. (**B**) HPLC chromatogram in supernatants of retina (negative control). (**C**) HPLC chromatogram in supernatants of retina instilled with FEN.

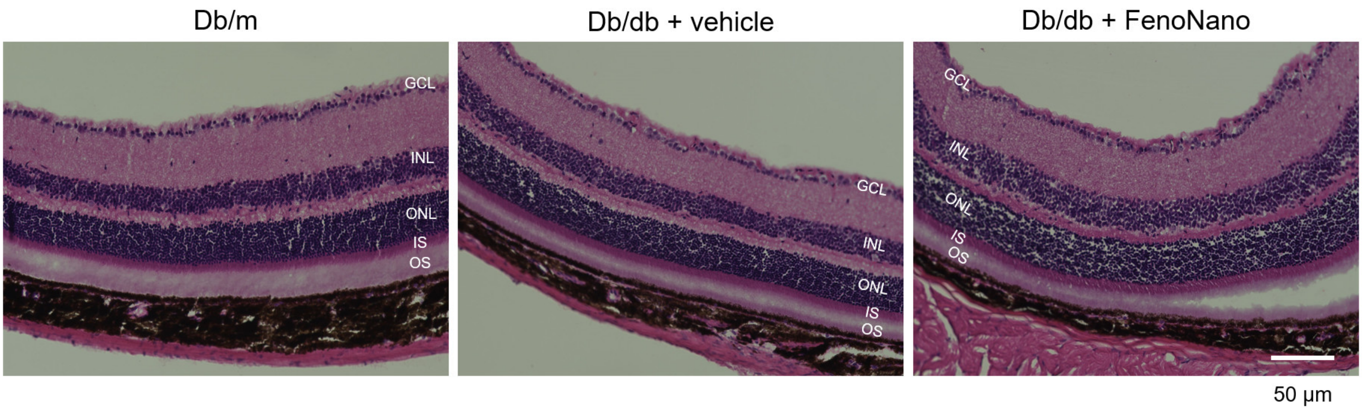

Figure S3. The HE stainings of the whole retina in non-diabetic control db/m, db/db with vehicle, and db/db with FenoNano eyedrop at 14 weeks of age.
